# Direct-to-Consumer Advertising of Prescription Drugs: What Can the Swiss Experience Tell Policy-Makers in the United States?

**DOI:** 10.7759/cureus.101400

**Published:** 2026-01-12

**Authors:** Omolayo T Umaru, Taiwo O Aremu

**Affiliations:** 1 Department of Pharmaceutical Care and Health Systems, College of Pharmacy, University of Minnesota, Minneapolis, USA; 2 Division of Environmental Health Sciences, School of Public Health, University of Minnesota, Minneapolis, USA; 3 Department of Pediatrics, Medical School, University of Minnesota, Minneapolis, USA; 4 Department of Clinical and Social Pharmaceutical Sciences, College of Pharmacy, Touro University California, Vallejo, USA

**Keywords:** cost-effectiveness, direct-to-consumer advertising, medicare spending, pharmaceutical marketing, pharmaceutical regulation, pharmacy practice, prescription drugs, public health policy, switzerland, united states of america

## Abstract

Direct‑to‑consumer advertising (DTCA) of prescription drugs refers to the promotion of prescription‑only medicines directly to patients and the general public through channels such as television, print, and online media, and it remains a contested practice. In this editorial, we examine the tension between the purported benefits of DTCA (greater disease awareness and patient engagement) and its documented harms, including distorted expectations, brand-driven demand, and higher drug spending. Using Switzerland's more restrictive approach to public promotion and its integration of cost-effectiveness into reimbursement decisions as a contrasting model, we argue that recent United States enforcement actions should be a starting point for structural reform rather than isolated crackdowns. We propose tighter limits on DTCA for higher‑risk drugs, stronger standards for risk communication, and closer alignment of advertising privileges with clinical and economic value, alongside support for pharmacists who manage advertising-driven requests. These changes would better align pharmaceutical promotion with patient safety, rational prescribing, and health-system sustainability.

## Editorial

Direct‑to‑consumer advertising (DTCA) of prescription drugs refers to the promotion of prescription-only medicines directly to patients and the general public through channels such as television, print, and online media, rather than solely via health professionals. Among high‑income countries, only the United States (US) and New Zealand permit product‑claim DTCA for prescription drugs, whereas in most other jurisdictions, including Switzerland, public advertising of prescription‑only medicines is prohibited. The US has nonetheless chosen to rely on disclosure rules and after‑the‑fact enforcement to keep this fundamentally promotional medium compatible with patient safety and rational prescribing.

The US experience with DTCA is not monolithically negative. Studies in cardiovascular prevention and other areas show that exposure to prescription drug advertising can be associated with increased intentions to seek care, discuss treatment options, and consider evidence‑based therapies among people at elevated risk [1]. Systematic reviews of DTCA and the patient-prescriber encounter similarly report that advertising often increases information‑seeking, generates appropriate requests for underused therapies, and can improve some patients’ perceptions of communication and involvement in decision‑making during visits [2,3]. Disease‑awareness messaging and patient‑facing information have helped destigmatize certain mental health conditions and encouraged individuals to talk to clinicians earlier in the course of illness. These are real and important benefits.

At the same time, DTCA operates within a commercial logic that is only partially aligned with clinical priorities. Advertisements typically highlight newer branded products rather than low‑cost generics. Patients who arrive at the clinic or at the pharmacy counter asking for a specific brand by name may be responding less to comparative effectiveness data and more to sophisticated marketing. This raises concerns about opportunity costs: advertising budgets are ultimately recovered through drug prices, and advertising‑driven demand can shift prescribing toward higher‑cost options. Over the past two decades, spending on medical marketing in the US has increased markedly; direct‑to‑consumer prescription drug advertising alone rose from about US$ 2.1 billion (11.9% of total medical marketing) in 1997 to US$ 9.6 billion (32.0%) in 2016 [4]. A Government Accountability Office (GAO) analysis of Medicare spending found that drugs with DTCA account for a disproportionate share of program expenditures, about US$ 324 billion out of US$ 560 billion in Parts B and D drug spending from 2016-2018, with two‑thirds of DTCA spending concentrated on just 39 brand‑name drugs [5]. More recently, an analysis of television DTCA showed that fewer than one‑third of the most heavily advertised drugs were rated as having high therapeutic value, and that roughly US$ 15.9 billion of US$ 22.3 billion in television advertising spending was devoted to products offering only low or modest added benefit [6]. Together, these findings demonstrate the fiscal implications of consumer‑directed promotion and its tendency to amplify demand for expensive, marginal‑benefit drugs.

The clinical effects of DTCA are similarly mixed. Polen and colleagues examined how advertising shapes patient behaviors and found a complex pattern: advertising could encourage care‑seeking and treatment initiation, but it also generated significant anxiety about adverse events and, in some cases, prompted patients to stop necessary medications [7]. A systematic review by Gilbody and colleagues found that DTCA increases patient demand for advertised medicines and the number of related prescriptions written but concluded that there was no clear evidence of improved population‑level health outcomes and raised concerns about inappropriate prescribing and cost [2]. More recent syntheses focusing on the patient-prescriber encounter also describe a dual effect: DTCA can facilitate earlier visits and more detailed discussions, yet prescribers report feeling pressured to honor some requests for lower‑value drugs and to spend additional time correcting misconceptions [3]. Patients with limited health literacy may be particularly vulnerable to dramatic risk warnings that lack clear context about absolute risks and the harms of untreated disease. Experimental work on branded prescription drug websites shows that when key risk information is moved from the homepage to a secondary page, patients recall significantly fewer risks and perceive the drug as safer, underscoring that the placement and salience of safety information can heavily influence risk understanding [8]. In everyday practice, clinicians and pharmacists must spend valuable time unpacking these messages, correcting misconceptions, and re‑establishing appropriate adherence.

Against this backdrop, Switzerland offers a strikingly different policy experiment. Switzerland serves as our primary comparator for three reasons. First, among high‑income countries that prohibit product‑claim DTCA for prescription‑only medicines, it has a particularly clear and well‑documented combination of strict limits on public promotion and explicit cost‑effectiveness criteria for reimbursement [9-11]. Second, it is home to major research‑based pharmaceutical companies and consistently ranks highly on health‑system performance and innovation, making it a “hard test” of whether strict promotional controls are compatible with therapeutic progress [9,11]. Third, focusing on one well‑characterized jurisdiction allows us to provide readers with a concrete, detailed contrast rather than a superficial tour of multiple DTCA‑banning countries.

In Switzerland, public advertising of prescription‑only medicines is prohibited. Swissmedic, the national therapeutic products agency, oversees medicinal product promotion, while the Federal Office of Public Health (FOPH) evaluates effectiveness, appropriateness, and cost‑effectiveness when deciding which medicines to list for reimbursement [9]. Advertising to the general public is limited to certain over‑the‑counter products under strict content rules and active regulatory surveillance.

Despite prohibiting prescription DTCA, Switzerland remains a global leader in pharmaceutical innovation. The country couples an advanced life‑sciences sector with strong performance on measures of health‑system quality and efficiency. Its regulatory architecture draws a clear line between scientific evidence, value assessment, and public promotion: new drugs must demonstrate effectiveness and, for reimbursement, value for money, while public advertising plays little role in shaping prescribing [9].

The contrast between the US and Swiss models raises two questions. First, is unconstrained DTCA necessary for innovation or patient empowerment? The Swiss example suggests the answer is no. Second, can US regulators rely indefinitely on case‑by‑case enforcement to manage the risks of DTCA? Recent developments suggest that they cannot.

In 2025, the US Food and Drug Administration (FDA) announced a major "crackdown" on deceptive drug advertising, reporting 1000s of letters directing the removal of allegedly misleading ads and approximately 100 cease‑and‑desist notices, alongside an intent to close the "adequate provision" loophole that has allowed broadcast advertisments to refer viewers elsewhere for full risk information [12]. These actions acknowledge that current safeguards have been insufficient: problematic advertisements still reach millions of viewers before corrective action is taken, and risk disclosures are often overwhelmed by positive imagery and reassuring narratives.

The recommendations that follow mix near‑term adjustments with longer‑term structural reforms. In the short term, US regulators could strengthen risk‑communication standards within the existing statutory framework and create payment and workflow supports for pharmacists who manage DTCA‑driven encounters. Over a longer horizon, more structural changes, such as restricting DTCA for higher‑risk or newly approved medicines and linking advertising privileges to evidence of clinical and economic value, would move US policy closer to the Swiss emphasis on cost‑effectiveness and patient protection while still being adapted to US legal and regulatory constraints. We outline four such directions below.

First, regulators could move beyond broad permission for prescription DTCA toward a more limited, conditional model. For example, DTCA could be restricted or prohibited for newly approved drugs with limited post‑marketing safety data, medicines with boxed warnings, or products in therapeutic classes with a history of serious safety issues. Such targeted limits would focus advertising where the risk-benefit balance is best established, rather than treating all prescription drugs as equally suitable for mass promotion.

Second, the FDA should strengthen risk‑communication standards to ensure that safety information is not simply present, but actually salient. This could include requirements for clear on‑screen "risk boxes" in television and digital advertisments, minimum font sizes, balanced pacing of spoken benefits and risks, and restrictions on distracting visuals during risk narration. Empirical work on patient responses to DTCA demonstrates that how risk information is presented matters as much as whether it is technically included [7], and randomized experiments with prescription‑drug websites show that keeping risk information prominent on main pages improves recall compared with burying it on secondary pages [8].

Third, the US should more explicitly integrate value considerations into the DTCA ecosystem. While the FDA's remit does not include pricing, other policy levers can promote transparency around cost‑effectiveness and encourage the use of high‑value therapies. In Switzerland, drugs are only reimbursed under compulsory basic insurance if they meet the Wirksamkeit, Zweckmässigkeit, Wirtschaftlichkeit (WZW) criteria of effectiveness, appropriateness, and cost‑effectiveness; the Federal Office of Public Health (FOPH) decides whether a product is included on the Specialities List and sets a maximum reimbursed price using external reference pricing and therapeutic cross‑comparison [9,10]. Each year the FOPH re‑evaluates roughly one‑third of the Specialities List against these criteria and mandates price cuts where economic efficiency is no longer met, for example, the 2023-2025 review cycle is expected to generate at least CHF 335 million in savings for compulsory health insurance, on top of approximately CHF 740 million saved in the 2017-2019 and 2020-2022 cycles [13]. The Swiss model, in which the FOPH evaluates cost‑effectiveness as a condition of reimbursement, shows that systematic assessment of value can coexist with innovation and access [9]. Recent evidence that the majority of US television DTCA spending is concentrated on drugs rated as low therapeutic value further underscores the need to reconnect advertising privileges with demonstrable clinical benefit [6]. This is particularly striking given that per‑capita retail pharmaceutical spending in the US (around US$ 1,700 in 2023) remains substantially higher than in Switzerland (around US$ 1,060) and well above the Organization for Economic Co-operation and Development (OECD) average (about US$ 760) [11]. In the US, linking privileged access to DTCA (for example, eligibility to advertise on broadcast television) to demonstrable improvements in clinical outcomes or cost‑effectiveness could better align commercial messaging with public health goals.

Finally, any serious reform must recognize the important role of pharmacists and other front‑line clinicians in mediating the effects of DTCA. When advertising prompts patients to request specific brands or to stop medications out of fear, it is pharmacists who often absorb the first wave of questions and conflicts. Survey and interview data synthesized in recent systematic reviews show that prescribers and pharmacists frequently report DTCA‑prompted requests, added time pressures in consultations, and occasional strain when they decline to prescribe advertised products [3]. Policy changes that tighten DTCA standards should be paired with mechanisms to support counselling time; for instance, reimbursable pharmacist consultations for complex medication decisions influenced by advertising.

The US and Swiss systems share a commitment to therapeutic innovation and patient care, but they diverge sharply on how much space pharmaceutical advertising should occupy in that landscape. In the US, spending on medical marketing grew from about US$ 17.7 billion to US$ 29.9 billion between 1997 and 2016, with DTCA for prescription drugs increasing from US$ 2.1 billion (11.9% of total medical marketing) to US$ 9.6 billion (32.0%) over the same period [4]. At the payer level, drugs with DTCA accounted for roughly US$ 324 billion of the US$ 560 billion Medicare spent on prescription medicines between 2016 and 2018, accounting for nearly 60% of total Part B and D drug spending [14]. Yet fewer than one‑third of the prescription drugs most heavily promoted on US television have been rated as offering high therapeutic value compared with existing treatments [6], and per‑capita retail pharmaceutical spending in the US (US$ 1,713 in 2023) remains substantially higher than in Switzerland (US$ 1,061) and the OECD average (US$ 766) [11]. Taken together, these data make it increasingly difficult to defend the status quo of broad, lightly constrained DTCA. As FDA and other agencies move to rein in deceptive advertisements, policymakers should seize the opportunity to reconsider the underlying model, not just its most egregious abuses.

In light of this evidence, we argue that US reforms should go beyond case‑by‑case enforcement to adopt structural safeguards inspired in part by the Swiss model, including stricter limits on prescription DTCA (particularly for higher‑risk products), stronger empirically informed standards for risk communication, and a more explicit linkage between advertising privileges and demonstrable clinical and economic value. A more restrained, value‑conscious approach to DTCA along these lines would better serve patients, clinicians, and the sustainability of the US healthcare system.
